# Video-Assisted Thoracoscopic Resection of Cervicomediastinal Masses Using Preoperative Virtual 3-Dimensional Imaging

**DOI:** 10.1016/j.atssr.2025.11.030

**Published:** 2025-12-20

**Authors:** Yi-Jia Lee, Kuan-Hsun Lin, Shao-Cheng Liu, Hsu-Kai Huang, Tsai-Wang Huang

**Affiliations:** 1 Division of Medical Education, National Defense Medical University, Taipei, Taiwan, Republic of China; 2 Division of Thoracic Surgery, Department of Surgery, Tri-Service General Hospital, National Defense Medical University, Taipei, Taiwan, Republic of China; 3 Department of Thoracic Surgery, Tri-Service General Hospital, School of Medicine, National Defense Medical University, Taipei, Taiwan; 4 Department of Otolaryngology-Head and Neck Surgery, Tri-Service General Hospital, National Defense Medical University, Taipei, Taiwan, Republic of China; 5 Department of Otolaryngology-Head and Neck Surgery, Tri-Service General Hospital, School of Medicine, National Defense Medical University, Taipei, Taiwan

## Abstract

This case report describes a 59-year-old woman with a residual cervicomediastinal thyroid mass after right thyroid lobectomy for papillary carcinoma. Because of its deep intrathoracic extension and proximity to major vessels, a purely cervical approach was not feasible. The operation was planned using high-resolution, contrast-enhanced computed tomography and preoperative 3-dimensional reconstruction to delineate vascular and anatomic relationships. Video-assisted thoracoscopic surgery enabled complete resection with uneventful recovery. This case highlights how 3-dimensional imaging can enhance surgical planning, precision, and safety in complex mediastinal procedures.

Intrathoracic thyroid masses constitute 5.8% of all mediastinal masses. Among these, cervicomediastinal masses are considered to have an absolute indication for surgical intervention.^1^ This case involves a residual mediastinal goiter after thyroid surgery that was managed with video-assisted thoracoscopic surgery (VATS) assisted by preoperative 3-dimensional (3D) reconstruction. This approach supported safer, anatomically informed intervention.

A 59-year-old woman with hypertension presented with a long-standing palpable mass in the right anterior neck. computed tomography (CT) of the neck revealed a 7 × 4 cm calcified lesion suggestive of an intrathoracic goiter. Results of fine-needle aspiration indicated a benign follicular nodule. Given more extensive calcification on the right and anticipated surgical difficulty on the left, right thyroid lobectomy was performed to preserve vocal cord function. Pathologic examination confirmed papillary thyroid carcinoma (pT1b[m] Nx, stage I).

Four months later, a follow-up ultrasound scan showed a persistent left-sided mass. Although the results of fine-needle aspiration were nondiagnostic, malignancy was confirmed postoperatively, prompting planned completion surgery. Chest CT revealed a 7.1-cm residual mediastinal mass with tracheal deviation and compressive features (Figure 1). To delineate anatomic relationships, a 3D reconstruction using Mimics software (version 21.0, Materialise) was created, demonstrating tumor compression of the aortic arch, right brachiocephalic trunk, and left common carotid artery (Figure 2).

**Figure 1:**
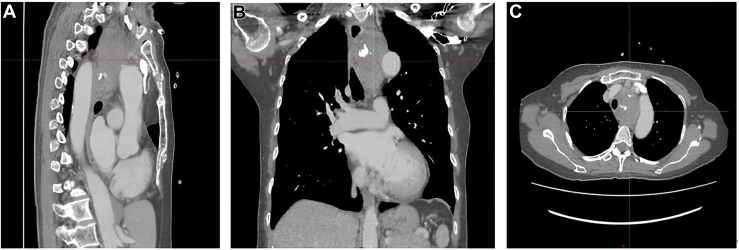
Contrast-enhanced computed tomography showing a residual cervicomediastinal thyroid mass (intrathoracic goiter) with mediastinal extension and mass effect on adjacent structures. (A) Sagittal view demonstrating the craniocaudal extent of the mass. (B) Coronal view highlighting the intrathoracic goiter. (C) Axial view demonstrating the lesion and its relationship to the trachea and surrounding mediastinal structures.

**Figure 2:**
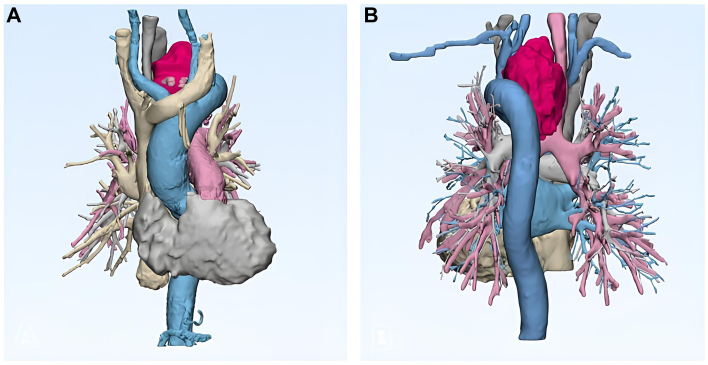
A 3-dimensional reconstruction of a mediastinal tumor using Materialise 3-matic software. The model shows bilateral enlargement of the tumor compressing the aortic arch, right brachiocephalic trunk, and left common carotid artery. (A) Anterior view. (B) Posterior view.

Given the presence of residual intrathoracic thyroid tissue, the patient underwent VATS surgery for mediastinal tumor resection and lymph node dissection (lymph node station 4R). The procedure was meticulously planned to use the preoperative 3D model, which provided detailed information on tumor size, local invasion, and vascular involvement. The surgical procedure involved reopening the transcervical incision and adding 3 thoracic ports at the right fourth to sixth intercostal spaces. Careful dissection preserved the trachea, azygos vein, superior vena cava, and aorta. The well-encapsulated mass was excised completely, and hemostasis was achieved with adjunctive agents.

Postoperatively, the patient was monitored in the intensive care unit and experienced only mild wound pain without any respiratory distress or vocal cord dysfunction. The final pathology report confirmed benign residual thyroid tissue. Results of pulmonary function tests, including lung volumes and diffusion capacity, were normal. She was discharged in stable condition. At 5-month follow-up, posteroanterior chest radiographic imaging confirmed good postoperative lung expansion with no signs of respiratory distress (Figure 3). Laboratory values were stable (thyroid-stimulating hormone, 0.07 μIU/mL; thyroglobulin, < 0.2 ng/mL; antithyroglobulin antibodies (IU/mL), negative). An iodine-131 whole body scan was scheduled for 1 year postoperatively to evaluate for potential residual disease. The Video illustrates the case overview, 3D modeling, and intraoperative VATS technique.

**Figure 3:**
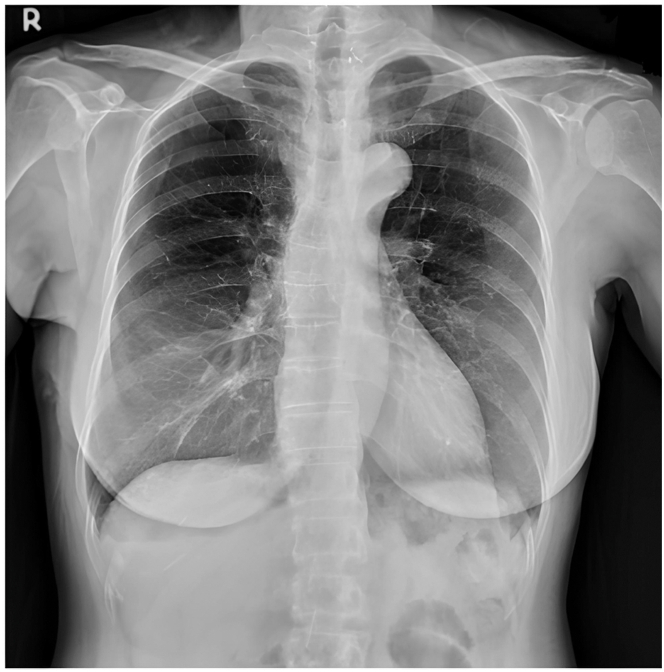
Postoperative chest roentgenogram (posteroanterior view) demonstrating no abnormalities in the right (R) thoracic cavity, with good lung expansion.

## Comment

In this case, completion thyroidectomy was indicated after confirmation of malignancy in the right thyroid lobe.^2^ However, the presence of extensive calcification, fibrotic adhesions, and proximity to major mediastinal structures rendered a transcervical approach unfeasible. A thoracic approach was therefore selected. Among available options—sternotomy, clavicular resection, thoracotomy, and VATS—the last option was preferred because of its minimally invasive nature and suitability for this tumor’s anatomic location.^1^^,^^3^

Surgical resection in the middle mediastinum is inherently challenging as a result of the limited operative field and the density of vital structures. Precise preoperative imaging is thus essential to minimize intraoperative risk. Whereas contrast-enhanced CT provides basic anatomic delineation, 3D reconstruction offers superior spatial orientation and improved visualization of tumor-vessel relationships.^4^ Unlike magnetic resonance imaging, which lacks detailed resolution of vascular and airway structures,^5^ 3D reconstruction supports operative planning, facilitates dissection, and enhances team communication. It may also assist in patient counseling regarding surgical risks.

Nonetheless, limitations exist. Cost, technical expertise, and institutional availability remain barriers to widespread implementation. Variation in software platforms and hardware requirements may affect reproducibility and integration into routine workflows. These factors should be considered when selecting cases for advanced preoperative visualization.

This case also provided several practical lessons. Combined cervical and thoracoscopic access is useful when a thyroid remnant extends across the thoracic inlet. Preoperative 3D reconstruction helps clarify the spatial relationships between the mass and major mediastinal vessels and supports safe operative planning. Careful identification and preservation of the recurrent laryngeal nerves and great vessels remain critical to preventing complications during resection.

In conclusion, this case highlights the role of 3D imaging in the preoperative assessment and surgical planning of cervicomediastinal thyroid masses requiring thoracic access. Integration of 3D reconstruction into VATS allowed precise evaluation of tumor extent and vascular anatomy, thereby facilitating safe resection with favorable postoperative outcomes. Although resource limitations may constrain its universal adoption, 3D imaging represents a valuable adjunct in complex thoracic procedures involving anatomically challenging lesions.

## Declaration of Generative AI and AI-Assisted Technologies in The Writing Process

During the preparation of this work, the authors used ChatGPT (OpenAI) to assist with language clarity and sentence refinement. After using this tool/service, the authors reviewed and edited the content as needed and take full responsibility for the content of the publication.
